# Histaminylation of glutamine residues is a novel posttranslational modification implicated in G-protein signaling

**DOI:** 10.1016/j.febslet.2012.09.027

**Published:** 2012-11-02

**Authors:** Jakob Vowinckel, Silke Stahlberg, Nils Paulmann, Katharina Bluemlein, Maik Grohmann, Markus Ralser, Diego J. Walther

**Affiliations:** a Max Planck Institute for Molecular Genetics, Ihnestrasse 73, 14195 Berlin, Germany; b Cambridge Systems Biology Centre & Dept. of Biochemistry, University of Cambridge, Sanger Building, 80 Tennis Court Road, Cambridge CB2 1GA, UK; c Department of Biology, Chemistry, and Pharmacy, Free University Berlin, Takustrasse 3, 14195 Berlin, Germany

**Keywords:** HA, histamine, PTM, posttranslational modification, TGM, transglutaminase, SRM, selective reaction monitoring, CTA, cysteamine, Transglutaminase, Histamine, Histaminylation, G protein, Posttranslational modification

## Abstract

Posttranslational modifications (PTM) have been shown to be essential for protein function and signaling. Here we report the identification of a novel modification, protein transfer of histamine, and provide evidence for its function in G protein signaling. Histamine, known as neurotransmitter and mediator of the inflammatory response, was found incorporated into mastocytoma proteins. Histaminylation was dependent on transglutaminase II. Mass spectrometry confirmed histamine modification of the small and heterotrimeric G proteins Cdc42, Gαo1 and Gαq. The modification was specific for glutamine residues in the catalytic core, and triggered their constitutive activation. TGM2-mediated histaminylation is thus a novel PTM that functions in G protein signaling. Protein αmonoaminylations, thus including histaminylation, serotonylation, dopaminylation and norepinephrinylation, hence emerge as a novel class of regulatory PTMs.

**Structured summary of protein interactions:**

**TGM2**enzymaticly reacts**CDC42** by enzymatic study (View interaction)

**TGM2**enzymaticly reacts**G α 01** by enzymatic study (View interaction)

**Pak3**physically interacts with **CDC42** by solid phase assay (View interaction)

**TGM2**enzymaticly reacts**G α q** by enzymatic study (View interaction)

**G α 1**binds to **Rgs4** by pull down (View interaction)

**CDC42**physically interacts with **Pak3** by pull down (View interaction)

## Highlights

► Histamine is covalently attached to protein-integrated glutamine residues. ► Transglutaminase II (TGM2) is catalyzing protein histaminylation in P815 mast cells. ► Histaminylation of a catalytic glutamine activates G proteins Gαq, Gαo1 and Cdc42. ► Histaminylation is a novel posttranslational modification belonging to the class of protein monoaminylations.

## 1 Introduction

Posttranslational modifications including phosphorylation, acetylation, methylation or glycosylation are essential in cellular signaling cascades and used to regulate protein activity [1,2]. Another, but less understood posttranslational modification is the attachment of serotonin, a biogenic monoamine, to glutamine residues [3,4]. Serotonin is transferred in a transglutaminase (TGM)-dependent manner to G proteins in aggregating mouse platelets [3]. Upon this discovery, several pathophysiological roles for serotonylation have been reported, including a function in diabetes and primary pulmonary hypertension, indicating that this modification plays an important biological role [5–12].

Interestingly, it has recently been shown that monoamine transfer by TGM is not limited to serotonin; also norepinephrine and dopamine were found to be TGM substrates in transamidation reactions [13,14]. Although no biological roles for dopaminylation and norepinephrinylation are yet known, these observations indicate that transfer of biogenic monoamines (= monoaminylation) could be representatives for a broad class of previously neglected posttranslational modifications.

In the present study, we address the biological function of another monoamine incorporation, histaminylation. Histamine (HA) plays an important biological role as neurotransmitter and in the inflammatory response [15]. In these cases, histamine signaling is induced by G protein coupled receptors, that are activated through binding histamine as non-covalent ligand [16,17]. However, although still lacking a recognized function, work in the 1960s–1980s also provided evidence for histamine incorporation into proteins [18–21].

Here we show that protein incorporation of histamine occurs in mast cells, and is catalyzed by transglutaminase II (TGM2). We identify glutamine residues in the catalytic core of the G proteins Gαq, Gαo1 and Cdc42 to be modified by histamine attachment, and provide evidence that this modification leads to their constitutive activation. Hence, histamine transfer to glutamine residues is a regulatory posttranslational modification. Targeting G proteins Gαo, Gαq and Cdc42, this novel modification is thus involved in mammalian signaling.

## 2 Materials and methods

### 2.1 Protein expression

Expression of TGMs was determined by PCR using the primers listed in Table 1. Constructs for the expression of N-terminally tagged GST and 6×His fusion proteins of Gαo1 (NP_059023), Gαq (NP_032165), Cdc42 (NP_001782), RGS4 (NP_058910 and Pak3BD (NP_032804 [AA 70–106]) were produced according to standard protocols. In brief, RNA from mouse tissue was isolated and reverse transcribed. Fragments corresponding to the respective small and heterotrimeric GTPases as well as effector protein domains were amplified by PCR. The respective fragments were introduced into the pGEM-T vector (Promega) and subcloned into the pGEX4T-1 (GE Healthcare) or pQE-40 (Qiagen). pGEX-p50RhoGAPΔ was a gift from A. Hall (MSKCC, New York [22]). For the expression of GST-WDTAGQERFR, a corresponding oligonucleotide was synthesized and cloned into the *Eco*RI/*Not*I sites of pGEX4T-1along side a linker (PASGGGAVNPASGGGGA). Recombinant fusion proteins were expressed and purified from *E. coli* BL21(DE3) competent cells with pREP4-groESL [23,24]. 6×His-Gαo1 was produced by the autoinductory expression procedure [25].

### 2.2 Monoaminylation assay

In vitro transamidation reactions were carried out as previously described [26,27]. rmTGM1, gpTGM2 and rhTGM3 were obtained commercially (Zedira), and 0.7 U rhTGM3 was activated with 0.2 μg Dispase II (Roche) and 50 mM Tris/HCl at pH 8.0 in a total volume of 10 μl at 30 °C for 20 min. For each protein, 200 pmol were mixed with 1 mM DTT, 10 mM CaCl_2_, Complete Protease Inhibitors without EDTA and the indicated concentrations of [^3^H]-labeled HA (GE Healthcare) or monodansylcadaverine (MDC) in 50 mM Tris/HCl, pH 8.0. Monoaminylation was initiated by the addition of 250 mU rmTGM1, 5 mU gpTGM2, 25 mU rhTGM3 and incubated for 30 min at 30 °C to give a total volume of 25 μl. To determine the extent of histaminylation, the reaction was terminated with 1 mg/ml BSA and 25% (v/v) PCA. After 2 h on ice, the precipitated protein was separated from unbound HA by filtration over GF-C filter disks and washed three times with ice cold washing buffer (100 mM PCA, 100 mM NaCl and 0.1% (v/v) Tween-20 in PBS). The radioactivity was measured with 5 ml ReadyProtein+ in a scintillation counter (Beckman). To determine incorporation of MDC, samples were subjected to SDS–PAGE, observed under UV light (BioRad GelDoc 2000) and stained using coomassie.

Culture of the mouse lymphoblast-like mastocytoma cell line P815 was as detailed previously [28]. Histaminylation of 1 × 10^6^ cell/well was determined as described [6], with TGM inhibition performed by applying 0.5 mM CTA where indicated.

### 2.3 Determination of GTPase activity

TGM2-dependent GTP hydrolysis of 6×His-Gαo1 or 6×His-Cdc42 was tested after histaminylation as described above followed by the addition of 5 mM MgCl_2_, 5 μM GTP and 0.1 μCi [α-^32^P]-GTP or [g-^32^P]-GTP (GE Healthcare) in a total volume of 30 μl. For thin layer chromatography, the sample was incubated at 37 °C for 45 min, terminated with 1% (w/v) SDS and analyzed on polyethyleneimine-cellulose plates with 1 M LiCl as the solvent [29]. For filter experiments, the sample was incubated at RT for 15 min, filtered over NC filter disks and washed three times with ice cold dilution buffer (50 mM Tris pH7.6, 50 mM NaCl, 5 mM MgCl_2_). The radioactivity was measured with 5 ml ReadyProtein+ in a scintillation counter (Beckman). GTPase effector binding activity was assayed using 6×His-tagged Cdc42 or Gαo1 and GST-tagged Pak3 Cdc42 binding domain (Pak3BD) or RGS4, respectively. For pull-down experiments, monoaminylation of 6 μM 6×His-Cdc42 or 2.5 μM 6×His-Gαo1 was conducted as described above with 200 μM HA and gpTGM2. The proteins were then preloaded with 500 μM GTP, GDP or GMP-PNP in the presence of 0.5 mM EDTA and subjected to GTP hydrolysis by the addition of 5 mM MgCl_2_ and in the case of Cdc42, 0.2 μM GST-p50RhoGAPΔ. After 15 min at 37 °C, 40 nM GST-Pak3BD or 2 μM GST-RGS4 were added together with 20 μl of Ni–NTA magnetic agarose beads (Qiagen) in ice-cold interaction buffer (50 mM NaH_2_PO_4_, 300 mM NaCl, 10 mM imidazole, 0.01% (v/v) Tween-20, 10% (v/v) glycerol) and inverted for at least 4 h at 4 °C. Unbound proteins were then removed by extensive washing. Effector binding was determined by immunoblotting and densitometry.

For experiments involving immobilized effectors, 10 μg/ml GST-Pak3BD was incubated on a MaxiSorp surface (Nunc) overnight. After extensive washing with water and blocking (5% milk powder in PBST, 30 min), 0.35 μM monoaminylated and nucleotide-preloaded 6×His-Cdc42 was added to the surface together with 0.2 μM GST-p50RhoGAPΔ in interaction buffer, and incubated for 30 min at 30 °C. While extensive washing and blocking steps were applied, GTPase binding was determined by immunodetection using 5×His-specific antibodies (Qiagen) and TMB (Thermo Scientific) by a colorimetric quantification.

### 2.4 Identification of monoaminylated peptides by selective reaction monitoring and LIT fragmentation

Purified GST-fusion proteins of Gαo1 and Gαq were incubated with or without TGM2 in the presence of HA. After blocking the reaction, proteins were reduced with dithiothreitol and tris-(2-carboxyethyl)-phosphine, alkylated with iodoacetamide, and digested with trypsin as previously described [30]. The digests were filtered with Amicon-Ultra 3k centrifugal filters (Millipore), vacuum dried and re-suspended in a water/acetonitrile/formic acid solution prior to analysis. Tryptic peptides were separated with a water to acetonitrile gradient at a flow rate of 300 nl/min on a Zorbax 300SB-C_18_ nanocolumn (Agilent) using a 2D Ultra nano HPLC (Eksigent). Structure-specific information was obtained on a QTRAP5500 (AB/Sciex) hybrid triple quadrupole/ion trap mass spectrometer that was equipped with a Nanospray III ion source (AB/Sciex) and coupled online to the nanoLC system. SRM (Q1/Q3) transitions corresponding to tryptic peptides containing the catalytic glutamine of Gαo1 and Gαq were calculated with MRM pilot 2.0 (Applied Biosystems) and Chemdraw (Cambridgesoft) and tested on unmodified (non-TGM exposed) digests to tune the instrument parameters. Afterwards, Q1/Q3 masses were adjusted for the expected mass shift in case of a histaminylation (Fig 2a,b). Fragment ion (MS/MS) spectra of the histaminylated and unmodified Gαo1 peptide were obtained on the QTRAP instrument (ion trap) operating in enhanced product ion (EPI) mode and targeting a precursor *m*/*z* of 493.3 for the histaminylated Gαo1 peptide and 446.2 for the unmodified doubly-charged Gαo1 peptide.

### 2.5 Statistical analyses

All data are presented as the means ± standard deviation, and *p* values are from two-tailed Student’s *t* tests. Values of *p* < 0.05 were considered as statistically significant.

## 3 Results

### 3.1 Histamine is incorporated into the mastocytoma proteome in a TGM- dependent manner

**Fig. 1:**
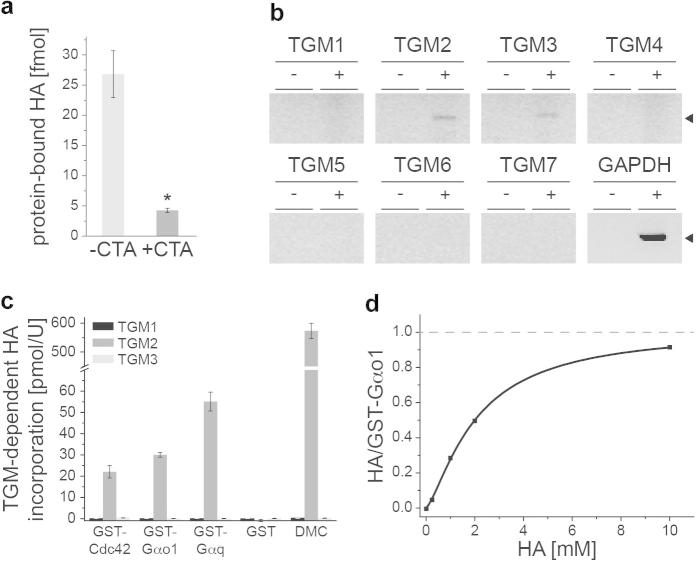
TGM2-dependent protein incorporation of histamine. (a) *Histamine added extracellularly is protein-incorporated*. P815 mastocytoma (1 × 10^6^) cells were treated with 1 μCi [^3^H]-histamine ([^3^H]-HA), with and without the transglutaminase (TGM) inhibitor cysteamine (CTA), proteins precipitated with PCA, and analyzed by scintillation counting. The [^3^H]-HA-treatment resulted in radioactive labeling of the protein fraction; transfer of the label was inhibited by CTA. (b) *TGM paralogues are expressed in mastocytoma cells*. RT-PCR experiments addressing TGM paralogues 1–7 from P815 cDNA. (c) *TGM2 transfers histamine to G proteins.* 8 μM GST, GST-Cdc42, GST-Gαo1, GST-Gαq or dimethyl caseine (DMC) were incubated for 30 min with 0.3 μM [^3^H]-HA in combination with TGM1, TGM2 or TGM3. Proteins were precipitated, filtered and analyzed by scintillation counting. (d) *Stoichiometry of Gαo1 histaminylation*. 1.6 μM GST-Gαo1 and 5 mU TGM2 were incubated for 60 min with differing amounts of [^3^H]-HA and assayed for radioactivity.

We found radioactivity in protein fractions obtained from a mastocytoma cell line (P815) that had been treated with tritium-labeled histamine ([^3^H]-HA) (Fig 1a). This indicates that HA added extracellularly is incorporated into the proteome, confirming previous reports [18–21]. Since it has been reported that histamine is a substrate for transglutaminases (TGM, E.C. 2.3.2.13, [20]) we speculated that member(s) of this enzyme class could catalyze the HA transfer. Indeed, treating P815 cells with the TGM inhibitor cysteamine (CTA) strongly reduced radiolabeling of the P815 protein extract (Fig 1a), providing evidence that transglutaminases could catalyze protein histaminylation, which would result in the formation of ω(γ-glutamyl)histaminyl residues.

Next, we performed RT-PCR experiments to establish whether transglutaminases are expressed in P815 cells. Expression of transglutaminase isozymes TGM2 and TGM3 was confirmed, whereas no PCR product was obtained for isozymes TGM 1, 4, 5, 6, and 7 (Fig 1b). Known targets of these TGMs are GTPases of the Rho and Rab family, such as Cdc42, and the heterotrimeric GTPases Gαq and Gαo1 [3,5–7,31,32] These proteins purified as GST fusion were incubated with either TGM1, TGM2 or TGM3 in the presence of [^3^H]-HA. No labeling was detected when TGM1 nor TGM3 were incubated with the substrates. However, TGM2 incubation resulted in radiolabeling of the G proteins and the positive control dimethyl caseine (DMC), but not GST alone (Fig 1c). Thus, TGM2 catalyzed a transfer of HA to the G proteins Gαo1, Gαq and Cdc42.

### 3.2 TGM2 transfers histamine to a single glutamine residue located within the catalytic core of Gαo1 and Gαq

**Fig. 2:**
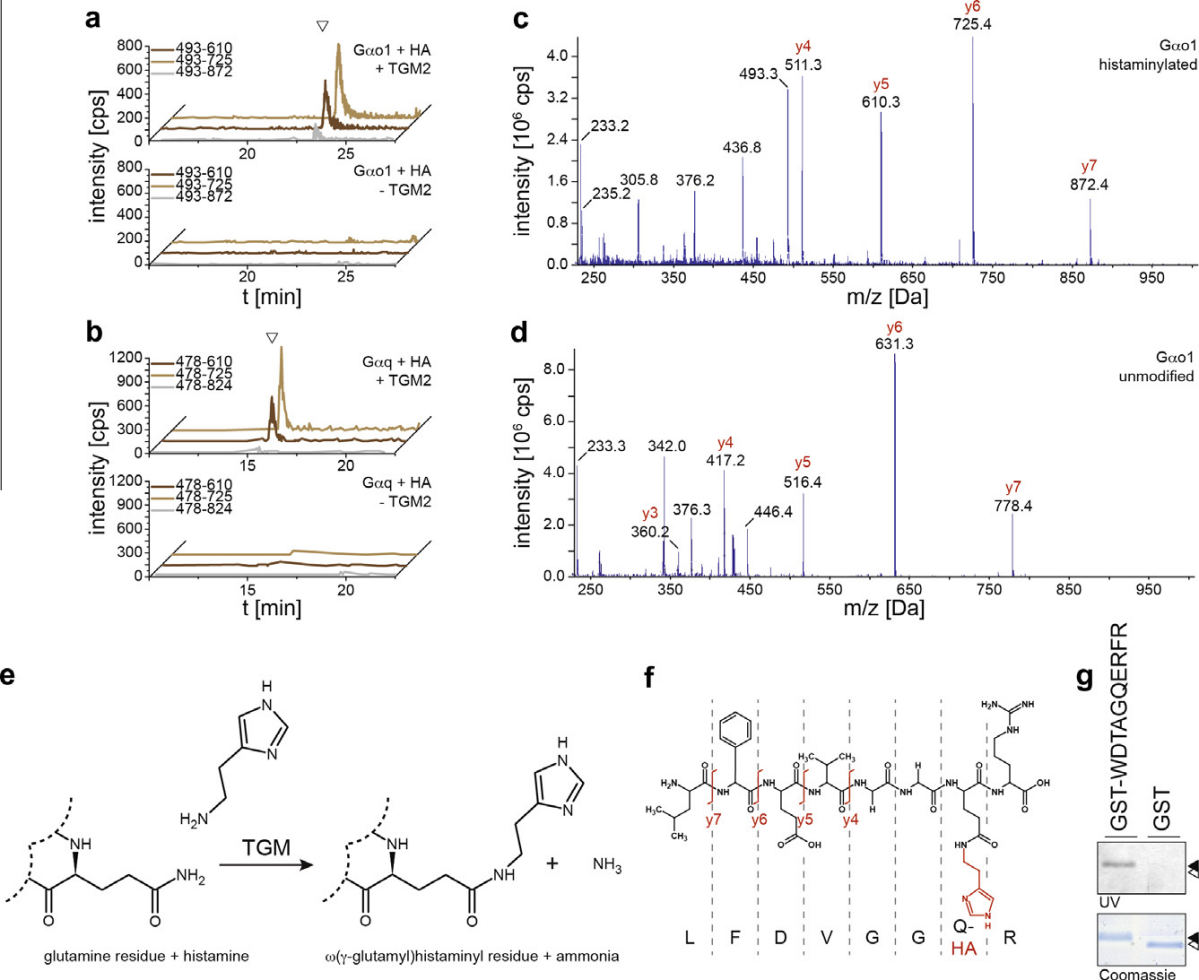
TGM2-dependent histaminylation of catalytic glutamines. (a) *SRM assays identify the Gαo1 catalytic-core glutamine as recipient of histaminylation.* GST-Gαo1 was incubated with 200 μM HA in the presence (upper panel) or absence (lower panel) of TGM2, digested with trypsin and analyzed by LC–MS/MS. Analyzed SRM (Q1/Q3) transitions (in *m*/*z*) are given in the legend. Please not that the corresponding Cdc42 peptide could not be analyzed as it exceeded the mass range (50–1200 Da *m*/*z*) of the mass spectrometer [39]. (b) Similar to (c), but analyzing Gαq. (c) MS/MS spectra of the histaminylated Gαo1 precursor peptide LFDVGGQ_HA_R and (d) of the unmodified Gαo1 precursor peptide LFDVGGQR. (e) *Posttranslational histaminylation of glutamine residues*. Transamidation of HA to a protein-bound glutamine residue, resulting in the formation of a ω(γ-glutamyl)histaminyl residue and release of ammonia. (f) *Schematic illustration* of the catalytic-core Gαo1 peptide containing a sole glutamine residue, modified with histamine. (g) *A linear GTPase core peptide confers TGM2 reactivity*. The Rab core peptide WDTAGQERFR, conserved in several GTPases, was fused with GST. Then GST or GST-WDTAGQERFR were incubated with TGM2 and the fluorescent TGM substrate MDC. Transamidation were determined using SDS–PAGE and UV detection.

To determine the stoichiometry of the histaminylation, we incubated GST-Gαo1 and TGM2 for 60 min with different [^3^H]-HA concentrations. After correction for unspecific binding, the HA/GST-Gαo1 ratio approached the saturation closely to 1, indicating histaminylation of a single residue (Fig 1d). We next used liquid chromatography/tandem mass spectrometry (LC–MS/MS) for localizing the modification. Using a nano LC- (nanoLC 2D Ultra, Eksigent) coupled hybrid triple quadrupole/ion trap mass spectrometer (QTRAP5500, AB/Sciex), we established selective reaction monitoring (SRM) assays [30,33] for tryptic digests of the G proteins Gαo1, Cdc42 and Gαq, taking into account a mass shift (+94) caused by potential histaminylations (Fig 2e). Peptides corresponding to the histaminylated catalytic center of Gαo1 (aa 199–206, Fig 2a) and Gαq (aa 203–210, Fig 2b) were detected by three SRM transitions each (upper panels). These SRM signals were specific to the enzymatic modification of Gαo1 or Gαq, as they were not detected in case HA and G proteins were incubated in the absence of TGM2 (lower panels).

The catalytic core tryptic peptide of Gαo1 peptide contains a single glutamine only (Fig 2f). Therefore, we also recorded MS/MS spectra of the TGM2 modified (Fig 2c) and non-modified (Fig 2d) peptides using the linear ion trap (LIT) mode of the Qtrap instrument. When compared to the MS/MS spectra of the corresponding unmodified peptide (Fig 2d), y-fragment ions of the modified peptide (Fig 2c) exhibited a *m*/*z* shift of +94, providing further indication for a histaminylation. Thus, both SRM assays and ion trap fragmentation indicate that TGM2 catalyzes the transfer of HA to the catalytic core peptide of G proteins Gαo1 (Q205) and Gαq (Q209).

Finally, we tested for sequence-specificity of TGM2-dependent histaminylation using a linear peptide (WDTAGQERFR) representing a common and highly conserved, G protein catalytic core. We generated a fusion protein of GST, a linker, and this peptide. When incubated with TGM2 and the fluorescent monoamine analogue MDC, the fusion protein was labeled, whereas GST alone (containing 6 glutamine residues), was not labeled (Fig 2f). Hence, monoaminylation of a synthetic G protein core peptide suggests that the specificity of TGM2 appears to be determined by a specific feature of the G protein catalytic core sequence.

### 3.3 Glutaminyl-histaminylation activates G proteins Gαo1 and Cdc42

**Fig. 3:**
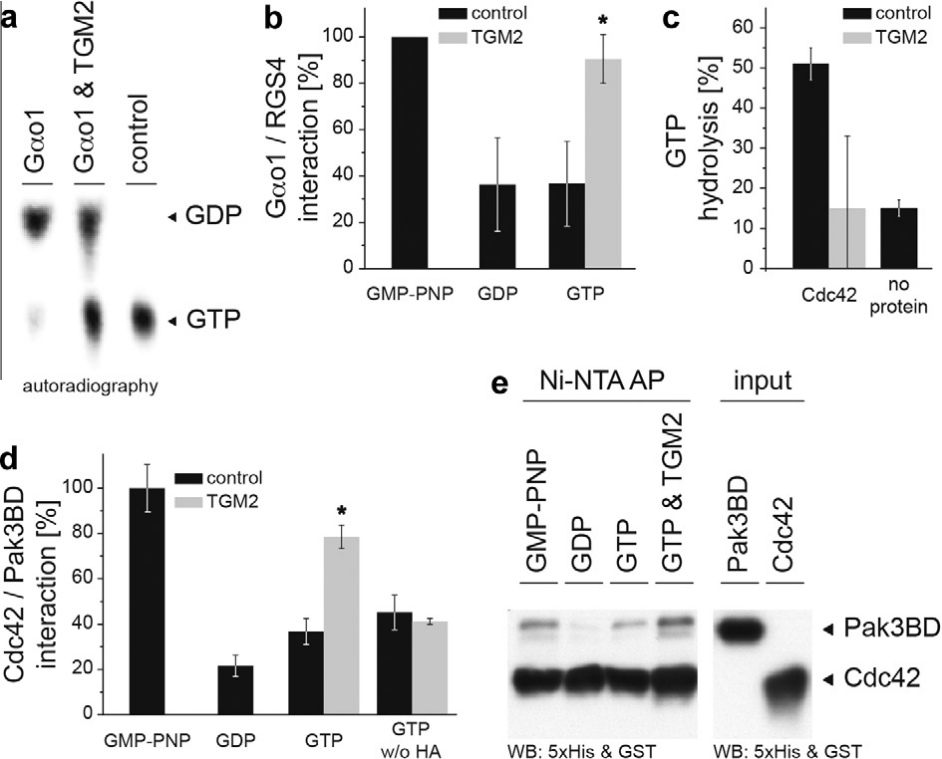
Histaminylated GTPases Cdc42 and Gαo1 show increased effector binding and decreased GTP hydrolysis. (a) *Histaminylation of G proteins prevents GTP hydrolysis.* Thin layer chromatography followed by autoradiography of [α-^32^P]-GTP incubated with native Gαo1, histaminylated (TGM2 exposed) Gαo1, and buffer. (b) *Histaminylation stabilizes a Gαo1/RGS4 complex*. 6×His-Gαo1, or TGM2/HA-exposed 6×His-Gαo1, was loaded with GMP-PNP, GDP and GTP and incubated with 2 μM GST-RGS4. The complex was immunoprecipitated (*n* = 5) and analyzed densitometrically. ^∗^*p* < 0.05. (c) *Histaminylation of the Rho GTPase Cdc42 prevents GTP hydrolysis*. 6×His-Cdc42 was exposed to TGM2/HA or HA alone, loaded with [γ-^32^P]-GTP, and GTP hydrolysis was induced by addition of MgCl_2_ and 0.2 μM p50RhoGAPΔ. After filtration, [γ-^32^P]-GTP was quantified using scintillation. (d, e) *Histaminylation stabilizes a Cdc42/Pak3 complex*. (c) 6×His-Cdc42 was pre-loaded with GMP-PNP, GDP and GTP, and incubated in multiwell plates with immobilized GST-Pak3BD in the presence of 0.2 μM p50RhoGAPΔ. Binding of 6×His-Cdc42 was strongly enhanced when the protein was pre-incubated with TGM2 and HA. (*n* = 3) ^∗^*p* < 0.05. (d) Pulldown of 40 nM GST-Pak3BD with 6 μM 6×His-Cdc42 pre-treated with GMP-PNP, GDP, GTP and GTP + TGM2/HA.

To test for biological function of the histaminylation, we tested whether this modification influences the activity of the G proteins. First, we used thin layer chromatography and studied the conversion of [α-^32^P]-GTP to [α-^32^P]-GDP in the presence of unmodified and histaminylated Gαo1. Whereas most GTP was hydrolyzed in the presence of naïve Gαo1, intensive GTP autoradiography was preserved in its histaminylated form (Fig 3a). Thus, histaminylated Gαo1 exhibits reduced GTPase activity, indicating that it is kept in its active (GTP-bound) conformation.

To verify an activation of Gαo1 by histaminylation, we studied the complex formation of Gαo1 with its binding partner RGS4. This protein is known to bind preferentially to active Gαo proteins [34]. Gαo1 was pre-loaded with GTP, GDP or GMP-PNP, a non-hydrolysable GTP analog leading to constitutive G protein activation. Then, the protein was incubated with recombinant RGS4, and the resulting protein complex was immunoprecipitated. Analyzed by western-blotting and densitometry, Gαo1 strongly bound RGS4 in the presence of GMP-PNP, whereas GDP- and GTP-preloaded Gαo1/RGS4-binding was significantly weaker (Fig 3b). Remarkably, histaminylated Gαo1 formed the RGS4 complex to the same extent as GMP-PNP pre-loaded Gαo1. Hence, histaminylation stabilizes the Gαo1/RGS4 complex, indicating that Gαo1 is constitutively activated by histaminylation.

We next analyzed the activity of the small GTPase Cdc42 as a function of histaminylation. As determined by [γ-^32^P]-GTP hydrolysis experiments, TGM2-catalyzed modification of the G protein leads to a strong decrease in catalytic activity compared to samples incubated with HA alone (Fig. 3c).

It has previously been shown that the binding affinity of Cdc42 and the Pak G protein binding domain increases upon activation of the G protein [35,36]. To provide additional evidence if histaminylation leads to activation of Cdc42, we thus studied the ability of Cdc42 to form a complex with the G protein binding domain of Pak3 (Pak3BD) [37,38] using a multiwell-based binding assay as described in the Materials and Methods section, and co-precipitation. Histaminylation of Cdc42 significantly increased its binding to Pak3BD in the multiwell-based binding assay (Fig 3c). This result was additionally confirmed by pulldown experiments, where a 6×His-Cdc42/GST-Pak3BD complex was purified using a Ni–NTA matrix and analyzed by western-blotting. When Cdc42 was histaminylated, or pre-loaded with the non-hydrolysable GTP analogue GMP-PNP, a significantly higher amount of Pak3BD was co-precipitated with Cdc42 (Fig 3e). Thus, catalytic-core histaminylation increases the GTP hydrolysis activity, and stabilizes protein interactions associated with the active state of Cdc42 and Gαo1. Taken together, these experiments indicate that the analyzed G proteins become constitutively active when histaminylated.

## 4 Discussion

It has been reported in 1961 that histamine is incorporated into proteins [17], and this discovery has been substantiated in the 1980s [21]. However, the function of protein-bound histamine has not been clearly defined until the present study. Here we provide evidence that histaminylation (histamine incorporation) functions as regulatory posttranslational modification*.* A selected group of GTPases, either known targets of TGM (Cdc42), or involved in regulation of vesicular HA transport (Gαo1 and Gαq), were shown to be histaminylated. These proteins become constitutively active upon this modification.

We have shown that histaminylation is catalyzed by transglutaminase TGM2, modifying only specific glutamine residues. Eventually, this transamidation leads to the formation of a ω(γ-glutamyl)histaminyl residue in the catalytic core of the small and heterotrimeric G proteins Cdc42 (modification at glutamine Q61), Gαo1 (Q205) and Gαq (Q209) (Fig 2e). (i) thin layer chromatography, (ii) quantification of their GTPase activity and (iii) increased binding to their effector domains indicated that these G proteins become constitutively active upon histaminylation. As the activity of the mast cell’s vesicular monoamine transporter (VMAT2), implicated in histamine uptake, depends on Gαq and Gαo [31], these findings imply that histaminylation functions in its auto-regulation. High cytosolic and/or vesicular HA concentrations could thus induce histaminylation of these GTPases, modulating the transporter’s activity and fine-tuning vesicular HA content.

However, these investigations do not allow concluding on the presence or absence of other targets for histaminylation. Follow up genome scale studies have now to clarify how broad the cellular function of this novel posttranslational modification is in total, and how many other proteins are functionally regulated by histaminylation. In this context, also other monoaminylations involving serotonin [3], and recently dopamine and *norepinephrin*e [13,14], have been shown to be transferred to glutamine residues in a TGM-dependent manner. Histaminylation is thus a member of a larger family of previously neglected posttranslational protein modifications (monoaminylations) that are implicated in mammalian signaling, whose spectra of biological function needs now to be defined [40].

## Figures and Tables

**Table 1:** Oligonucleotides for assessing of transglutaminase expression by RT-PCR.

Transcript	Orientation	Sequence
mTGM1	forward	5′-GTTTGAATATGATGAGCTGATTGTG-3′
	reverse	5′-TACTATGGAACAGAAGCACAGATTG-3′
mTGM2	forward	5′-GCCAACCACCTGAACAAACT-3′
	reverse	5′-CTTGATTTCGGGATTCTCCA-3′
mTGM3	forward	5′-CTGGAGAAGATCTGAATTTCATTGT-3′
	reverse	5′-AATGTCTTCTTCAAACTGTCCAAAG-3′
mTGM4	forward	5′-AGCAGAGTACATCCTTAATGACACC-3′
	reverse	5′-CCAGGTAGATGTCTACTGTGAGGTT-3′
mTGM5	forward	5′-TTCTGGAGAATATGAAGAAGGACAC-3′
	reverse	5′-TTTAGGAACACCTCTCTCTCTTGAA-3′
mTGM6	forward	5′-CAGCTCACAGTTCCCAGACA-3′
	reverse	5′-GAGTTACCTGGGCTGAGCTG-3′
mTGM7	forward	5′-GGGAGTGGCCTCATCAATGG-3′
	reverse	5′-CCTTGACCTCACTGCTGCTGA-3′
GAPDH	forward	5′-ACCACAGTCCATGCCATCAC-3′
	reverse	5′-TCCACCACCCTGTTGCTGTA-3′
